# Editorial: Teaching and training in psychophysiology and neuroscience

**DOI:** 10.3389/fpsyg.2025.1616546

**Published:** 2025-11-06

**Authors:** Erik M. Benau

**Affiliations:** Department of Psychology, SUNY Old Westbury, Old Westbury, NY, United States

**Keywords:** mentorship, early career academics, inclusive pedagogy, neuroscience training, STEM outreach

This Research Topic was inspired by the growing recognition that neuroscience education and engagement are extending well beyond the confines of traditional lecture halls and laboratories (Ramirez, 2020; Ramos and Rivera-Rodriguez, 2022). In the years leading to the proposal of this Research Topic, researchers and educators took bold steps to integrate neuroscience into K−12 outreach (Chang et al., 2021; Deal et al., 2014), adaptive technologies (Jangwan et al., 2022), sports training (Seidel-Marzi and Ragert, 2020), and inclusive learning environments (Dotson and Duarte, 2020; Harrington, 2022; Immordino-Yang and Damasio, 2007), among other areas (Eagleman, 2013; Heagerty, 2015). The aim of this Research Topic was to capture that momentum by assembling and disseminating work that promotes new approaches to training, mentorship, and pedagogy in neuroscience and psychophysiology. More specifically, when this Research Topic was proposed in 2022, neuroscience education—and education more broadly—was at an inflection point. The academic landscape was still reeling from the disruptions of the COVID-19 pandemic, and researchers across disciplines were adapting to new modes of public engagement, digital fatigue, and institutional constraints. Simultaneously, growing movements for equity, accessibility, and inclusion—within neuroscience and beyond—were challenging traditional models of outreach and participation (Dotson and Duarte, 2020; Harrington, 2022; La Scala et al., 2023).

Although the acute crises of that period have subsided in some ways, the residual effects of that time continue to shape how we teach, learn, and connect (Imran et al., 2023). Efforts to expand access also face increasing resistance, with some recent gains being rolled back and others encountering outright hostility. In recent months, in early 2025, the United States faced unprecedented cuts to science and education funding that disproportionately targeted diversity- and inclusion-related initiatives, including training programs. These reductions have already led—or may soon lead—to the displacement of numerous early-career scientists and trainees, many of whom work in under-resourced or equity-focused settings. These are the very individuals this Research Topic was intended to support; their uncertain futures speak to the urgency of continued investment in inclusive neuroscience training. The impact of these cuts will likely be felt for years and may slow progress in building a more inclusive and accessible neuroscience community. Nevertheless, the need remains urgent to develop and share innovations in neuroscience education—approaches that support critical thinking, inclusive practice, and public engagement. In this context, the urgency of developing neuroscience education that is both innovative and responsive is ever more apparent. Efforts to support critical thinking, inclusive practice, and public engagement remain essential not only for training future scientists, but also for sustaining a field that continues to face social and political pressures.

This Research Topic's call for studies welcomed contributions on formal instruction, along with informal and often under-discussed aspects of training. These include mentorship, interdisciplinary collaboration, and building sustainable research environments. The final Research Topic is more focused than originally envisioned, though this scope offers a certain clarity. Rather than cataloging the full range of emerging practices, the included articles offer insights into four key areas: early education and pipeline development, applied performance monitoring, cognitive accessibility, and the role of motivation in learning. These four contributions highlight the breadth of contexts in which neuroscience and psychophysiology are taught, applied, and communicated. They also point toward future directions for how neuroscience and psychophysiology may be taught and used. Below, each article is outlined with its key contributions.

## Early education and pipeline development

Minen et al. presented a critical systematic review of K−12 neuroscience and neurology outreach programs. Drawing on 23 studies, the authors highlight the range of educational strategies used to introduce students—particularly those from historically underrepresented backgrounds—to neuroscience concepts and careers. The review identified the strengths of interactive programming and partnership-based delivery models, while also noting a lack of long-term outcome data and equity-focused assessments. The synthesized findings illustrate the importance of early exposure and structured outreach in cultivating a diverse future neuroscience workforce.

## Applied performance monitoring

Yang et al. examined the usefulness of session rating of perceived exertion (sRPE) as a proxy for physiological training load (TRIMP) in elite cross-country skiers. The authors found that sRPE correlates well with TRIMP—particularly during low-intensity training—and may serve as a viable substitute when heart rate data are unavailable. This work demonstrates how cognitive-affective metrics can be used in high-performance training and highlights the growing role of applied neuroscience in settings not typically considered educational.

## Cognitive accessibility and assistive tech

Chan et al. presented a pilot study that assessed whether performance on the Eriksen Flanker Task could help identify students with disabilities who could benefit from visual accommodative technologies. Using a triple-blind design with students both with and without disabilities, the study demonstrated its sensitivity to attentional differences across groups. The authors suggest that these tools could support the more accurate identification of students in need of visual accommodations. This approach may contribute to improving accessibility in higher education through the use of psychophysiological methods.

## Motivation, emotion, and learning

Wang et al. explored how emotional motivation—both its intensity and direction—interacts with task load to shape cognitive flexibility. Across three experiments using arithmetic estimation tasks, the authors found that affective states influenced strategy use differently depending on cognitive demands: low-intensity motivation supported flexibility under high task load, whereas avoidance-based motivation appeared more helpful in low-demand conditions. These findings highlight the role of affect in learning and suggest possible applications for tailoring instruction to emotional contexts.

## Summary

Together, these contributions highlight the value of purposeful mentorship, creative pedagogy, and transdisciplinary innovation in shaping the next generation of scholars, educators, and practitioners. From early outreach and performance monitoring to individualized learning support and emotion-informed instruction, each paper offers a unique entry point into neuroscience training and engagement. Collectively, they provide practical illustrations of how neuroscience education can—and should—evolve. As educational demands shift and the future grows increasingly uncertain, the call for a more inclusive, accessible, and socially relevant approach to training neuroscientists has never been more urgent.
